# METRNL mitigates oxidative stress and inflammatory drawbacks in ovalbumin/lipopolysaccharide-induced allergic airway diseases via the IKK/IκB/NF-κB signaling pathway

**DOI:** 10.1007/s00210-025-04070-6

**Published:** 2025-04-17

**Authors:** Suzan A. Khodir, Anwaar M. Shaban, Eman Sweed, Noha M. Abd El-aziz, Basma Abdelnaby Mostafa, Asmaa A. Abdel Latif, Mai M. El-Kalshy, Eman I. Elgizawy

**Affiliations:** 1 Medical Physiology Department, Faculty of Medicine, Menoufia University, Menoufia, Egypt; 2 Medical Physiology Department, Menoufia National University, Menoufia, Egypt; 3 Clinical Pharmacology Department, Faculty of Medicine, Menoufia, Egypt; 4 Clinical Pharmacology Department, Menoufia National University, Menoufia, Egypt; 5 Anatomy and Embryology Department, Faculty of Medicine, Menoufia, Egypt; 6 Medical Biochemistry and Molecular Biology Department, Faculty of Medicine, Menoufia, Egypt; 7 Medical Biochemistry and Molecular Biology Department, Menoufia National University, Menoufia, Egypt; 8 Public Health and Community Medicine Department, Faculty of Medicine, Industrial Medicine and Occupational Health Specialty, Menoufia University, Menoufia, Egypt; 9 Department of Chest Diseases, Faculty of Medicine, Menoufia University, Menoufia, Egypt

**Keywords:** METRNL, Oxidative stress, Airway inflammation, NF-κB

## Abstract

This study aimed to examine the potential impacts of METRNL as an antioxidant and anti-inflammatory through IκB kinase/inhibitor of nuclear factor-kappa B/nuclear factor-kappa-light-chain signaling pathway on many biomarkers and lung structure in rats with bronchial asthma induced by ovalbumin/lipopolysaccharide (OVA/LPS). Forty rats were randomly divided into four equal groups: control group, vehicle group, diseased (OVA/LPS) group OVA 2.5 ml/kg intratracheal installation/LPS 1.5 mg/kg intraperitoneally, and treated (OVA/LPS + METRNL) group, METRNL at a dose of 2 mg/rat/day IV. After 4 weeks, plasma and lung tissues were analyzed to assess oxidative stress inflammatory markers. Additionally, a histological assessment was conducted on lung tissues. Bronchial asthma was confirmed when increased levels of total serum IgE, total cell count, neutrophils, eosinophils, macrophages, and lymphocyte counts in the BAL fluid were observed. Moreover, OVA/LPS resulted in a reduction in levels of superoxide dismutase (SOD) while raising levels of malondialdehyde (MDA). Furthermore, it elevated concentrations of plasma inflammatory mediators, including tumor necrosis factor-alpha (TNF-alpha), interleukin 17 (IL-17), and transforming growth factor beta (TGF-β). The protective effects of METRNL were analyzed. The observed impacts are believed to result from the drug’s anti-inflammatory and antioxidant properties and its action on the IKK/IκB/NF-κB signaling pathway. This investigation indicates that METRNL treatment positively improved rats’ biochemical and histological aspects of OVA/LPS-induced airway allergic inflammation.

## Introduction

According to the WHO (2003), allergic asthma is a long-term inflammatory respiratory illness brought on by an immune-mediated hypersensitivity reaction (Périz et al. 2020). This prevalent chronic noncommunicable disease affects approximately 270 million people worldwide (James et al. 2018). Airway inflammation is driven by various underlying factors, including airflow restriction and fluctuating respiratory symptoms (Papi et al. 2018). Although there is no cure, medication can manage the symptoms (Périz et al. 2020). While type II–driven inflammation plays a significant role in allergic asthma, other pathogenic mechanisms contribute to its complex pathophysiology (Agache 2019). Despite this, asthma remains underdiagnosed and, as a result, undertreated, which negatively impacts the quality of life and imposes a burden on communities, families, and nations (Rehman et al. 2018).

The newly secreted protein, meteorite-like, or METRNL, has pleiotropic effects on immunology, metabolism, and inflammation. A previous study on this hormone (Li et al. 2023) focused on its role in regulating glucose homeostasis and energy expenditure. Its numerous beneficial roles in regulating inflammatory immunity include activating macrophages, promoting angiogenesis, tissue remodeling, bone development, and preventing dyslipidemias (Chouchani and Kajimura 2019). A comprehensive study is required to determine the significance of this unique protein biomarker as a potential therapeutic agent in allergic conditions.

METRNL is a released protein that can be utilized as a biomarker and treatment for different diseases. It plays an essential role in physiological and pathological processes, providing epigenetic evidence of METRNL’s involvement in asthma improvement molecular mechanisms. It may hinder dendritic cell (DC) development and antigen presentation, thereby reducing type II inflammatory responses. Recombinant METRNL injection dramatically reduced T-cell proliferation (Gao et al. 2022).

Caspase-3 is a member of the caspase family (cysteine-aspartic proteases), a group of proteolytic enzymes known for their key role in the execution of apoptosis and its regulation. While primarily involved in apoptosis, caspase-3 can also influence inflammation through various signaling pathways (Zakariah et al. 2022).

The IκB kinase (IKK) complex, composed of the kinases IKKα and IKKβ, is a crucial regulator of all inducible nuclear factor-kappa-light-chain protein family of transcription factors (NF-κB) signaling pathways (Solt and May 2008).

NF-κB regulates the inducible expression of several genes that encode essential proteins involved in immunity, inflammation, and cell survival (Gilmore 2006). NF-κB is activated by various stimuli, including innate immune receptor ligation, antigen-receptor engagement, and pro-inflammatory cytokines like tumor necrosis factor (TNF-α) and interleukin 17 (IL-17) (Hayden et al. 2006).

Caspase-3-mediated p65 cleavage is believed to suppress NF-κB-mediated antiapoptotic transactivation in cells undergoing apoptosis. The fragment generated by Caspase-3 cleavage interferes with ribosomal protein S3, an NF-κB “specifier” subunit (Wier et al. 2015).

IKKβ is specifically proteolyzed by Caspase-3 during TNF-α-induced apoptosis. This proteolysis eliminates its enzymatic activity, interferes with IKK activation, and enhances TNF-α-mediated cell death. TNF-α-induced apoptosis requires caspase-mediated proteolysis of IKKβ (Dondelinger et al. 2015).

As the number of disorders involving the IKK/NF-κB system continues to rise, and this pathway is shown to be a primary mediator in many diseases, the importance of IKK/NF‐κB signaling in physiology and pathology is continually expanding (Tilstra et al. 2011). This work proposes a novel therapeutic approach for allergic asthma using METRNL. METRNL acts through the IKK/NF-κB pathway, with relatively limited pharmaceutical intervention in human diseases.

## Material and methods

A total of 40 mature male Wistar albino rats, weighing between 130 and 150 g, were obtained from the National Research Center (NRC) colony in Egypt. The sample size was calculated using G*Power software (version 3.1.9.7; Heinrich Heine University, Düsseldorf, Germany) with a power of 80% and a 95% confidence interval. The rats were housed in controlled conditions, which included a standard commercial pellet diet, unlimited access to water, a temperature of 22 ± 2 °C, 60% humidity, and a regular photoperiod (12-h light/dark cycle). The present investigation was conducted in accordance with regulations and relevant guidelines and was approved by the Faculty of Medicine Menoufia University Ethics Committee (IRB no. 8/2024BIO 13–1). The research methodology also adhered to the National Institutes of Health’s guidelines for the care and use of laboratory animals. The ARRIVE guidelines were followed in this animal experiment (Percie du Sert et al. 2020).

### Drugs and chemicals

Ovalbumin (OVA), derived from hen egg whites, was obtained from Sigma-Aldrich (St. Louis, USA) as a vial containing 1 g of lyophilized powder with a minimum albumin content of 90%. One gram of powdered aluminum hydroxide (Al(OH)₃) was also purchased from Sigma-Aldrich. *Escherichia coli* serotype 0111 lipopolysaccharide (LPS) was acquired from Sigma-Aldrich as a white-yellow lyophilized powder. METRNL, a product of Adipogen (San Diego, CA, USA), was dissolved in PBS. All additional chemicals and solvents used in the study were of the highest purity and were supplied by Sigma-Aldrich.

### Inducing an experimental model of allergic airway inflammation *(*Elaidy et al. 2018*)*

An animal model that precisely mirrored severe exacerbations of bronchial asthma seen in humans, specifically in terms of allergic airway symptoms, was created as a result of administering OVA + LPS treatments (Kumari et al. 2015). Following the protocols previously described by Dong et al. and Kumari et al., with adjustments to the frequency of challenge, a newly produced OVA solution was utilized prior to each administration (Dong et al. 2014; Kumari et al. 2015). For intraperitoneal sensitization, OVA (1 mg) adsorbed on aluminum hydroxide gel (20 mg) in a final volume of 2.5 mL/kg was utilized on days 0, 7, and 14. A challenge was conducted by administering 1.1% OVA dissolved in normal saline (200 µg) using intratracheal instillation on days 21, 23, 25, and 27 (Dong et al. 2014). LPS at a dosage of 1.5 mg/kg was dissolved in normal saline and administered intraperitoneally 1 h before each OVA dose on days 7, 21, 23, 25, and 27, as described by Haddad et al. (2001) and Kumari et al. (2015).

### Experimental protocol

Ten rats were randomly assigned to each of the four groups:

- Control group: This group included rats without bronchial asthma, airway inflammation, or medication administration.
- Vehicle group: The rats in this group were sensitized and challenged with aluminum hydroxide and normal saline, respectively, following the same protocols used for inducing airway inflammation and bronchial asthma. From days 15 to 28, the rats received 1 mL of PBS intravenously each day.
- Ovalbumin/lipopolysaccharide-induced bronchial asthma and airway inflammation (OVA/LPS) group: This group included rats exhibiting airway inflammation and bronchial asthma. From days 15 to 28, the rats were administered 1 mL of PBS intravenously each day.
- Ovalbumin/lipopolysaccharide-induced bronchial asthma and airway inflammation (OVA/LPS + METRNL) group treated with METRNL: This group included rats with bronchial asthma and airway inflammation. They received METRNL intravenously (2 μg/rat/day) dissolved in 1 mL of PBS for 14 days, starting from day 15 (Li et al. 2015; Jung et al. 2018).

### Blood sampling and total serum immunoglobulin E assay

At the end of 4 weeks, blood samples were collected from anesthetized rats by accessing the retrobulbar venous plexus. The serum was isolated by centrifuging the blood at 323 g for 20 min at 4 °C. The samples were then stored at − 80 °C for future analysis of total serum immunoglobulin E (IgE) levels. Total serum IgE levels were measured using a rat IgE enzyme–linked immunosorbent assay (ELISA) kit (ab157736; Abcam®, UK) and an automated ELISA reader (Metertech, M960), following the manufacturer’s instructions. Briefly, serum was added to the microtiter wells coated with IgE antibodies. Next, IgE antibodies labeled with conjugate were introduced. The conjugate bound immunologically to the IgE in the wells, resulting in the IgE molecules being sandwiched between the solid phase and the enzyme-linked antibodies.

### Quantification of leukocyte populations in bronchoalveolar lavage fluid (BALF)

After sacrifice, each rat’s trachea was cannulated, and three bronchoalveolar lavages (BALs) were performed using 5 mL of PBS. The BAL fluid was centrifuged at 81 g for 10 min at 4 °C. Following centrifugation, the cell pellet was resuspended in 1 mL of PBS and centrifuged again for 10 min at 81 g at 4 °C. Wright-Giemsa stain was applied for cell staining. A hemocytometer was then used to count the total and differential leukocytes with 10 μL of the solution (Guo et al. 2013)**.**

### Lung sample collection and processing

After collecting the BAL fluids, the lungs were washed with ice-cold PBS. The right lung was divided into two sections, frozen dry in liquid nitrogen, and stored at − 80 ℃ for subsequent laboratory tests and RNA extractions. The left lung was processed for histopathological and immunohistochemical analyses.

#### Preparation of lung tissue homogenates

A portion of each left lung has been thawed, weighed, and homogenized in 4 mL of chilled PBS. The resulting supernatants were analyzed to quantify TNF-α, IL-17, and TGF-β using rat-specific ELISA kits: the Rat Tumor Necrosis Factor-Alpha ELISA Kit (Cat. No. ERT2010-1 Assaypro LLC, Saint Charles, MO, USA), rat IL-17 ELISA kit (Cat. No. MBS164772, My BioSource, Inc., San Diego, USA), and rat TGF-β1 ELISA kit (Cat. No. CSB-E04727r, CUSABIO, Houston, TX, USA), following the manufacturer’s protocols. Lung malondialdehyde (MDA) and lung superoxide dismutase (SOD) were assessed using colorimetric kits from the Biodiagnostic Company (Giza, Dokki, Egypt).

#### Quantitative gene expression assay using reverse transcriptase polymerase chain reaction (RT-PCR)

The mRNA expression levels of IκB-α, IKK-β, and NF-κB in the lung tissue were quantified using RT-PCR. Total RNA was extracted from the tissues using TRIzol reagent (Invitrogen, Carlsbad, CA, USA) following the directions provided by the manufacturer’s protocol. The extracted RNA was stored at − 80 ℃ until further use.

For reverse transcription, complementary DNA (cDNA) was synthesized using ThermoScript™ RT reagent kits (Invitrogen). The cDNA was then amplified using SYBR Green Mix kits (Stratagene, USA) in polymerase chain reaction (PCR) assays. Amplification curves were analyzed to obtain cycle threshold (Ct) values. Glyceraldehyde-3-phosphate dehydrogenase (GAPDH) was used as the reference gene.

Data analysis was performed with the 7500 ABI PRISM system (Applied Biosystems, USA) using software version 2.0.1. The relative expression levels of IκB-α, IKK-β, and NF-κB were calculated using the comparative ΔΔCt method.

Primer sequences:

1. IKK-β: 
  (a) Forward: 5′-ACCAGAATCCGGGAAGACACAG-3′
  (b) Reverse: 5′-AGACGAGATCCATGTCCAGTGTG-3′
2. IκB-α:
  (a) Forward: 5′-TGGCCAGTGTAGCAGTCTTGAC-3′
  (b) Reverse: 5′-ATCAGCACCCAAAGTCACCAAG-3′
3. NF-κB-p65:
  (a) Forward: 5′-CACAGATACCACTAAGACGCACC-3′
  (b) Reverse: 5′-AGTCCTTCCCCACAAGTTCATG-3′
4. GAPDH (reference gene):
  (a) Forward primer: 5′-TATGACTCTACCCACGGCAAGT-3′
  (b) Reverse Primer: 5′-ATACTCAGCACCAGCATCACC-3′

### Histopathological studies

Lung tissue sections were obtained from all experimental groups for histopathological evaluation. These sections were fixed in 10% neutral buffered formalin (pH 7). Following fixation, the tissues were cleaned with xylol, dried with ethyl alcohol, and then embedded in paraffin. Hematoxylin and eosin were used to stain slices that were 4 µm thick.

Hematoxylin and eosin (H&E) was used to assess morphological changes in the lung tissue (Bancroft and Gamble 2008; Elkerdasy and Mousa 2021).

For immunohistochemistry, the lung paraffin Sects. (4 μm) were exposed to 3% hydrogen peroxide at room temperature for 10 min to block endogenous peroxidase activity. After rinsing with PBS, the sections were incubated with BCA solution for 30 min to prevent nonspecific binding. The sections were then probed overnight at 37 ℃ with primary antibodies: anti-caspase-3 (1:100 dilution, Elabscience Corp., Wuhan, China) for caspase-3-stained sections, and NF-κB (monoclonal, 1:200 dilution, Abcam) for NF-κB-stained sections.

Following primary antibody incubation, the sections were treated with a species-specific secondary antibody conjugated to peroxidase at room temperature for 30 min (Zhou et al. 2020). The staining intensities of caspase-3 and NF-κB were quantified using ImageJ software, with analysis performed at a magnification of 40 × (Nofal et al. 2019)**.**

### Statistical analysis

SPSS software version 27 (SPSS Inc., Chicago, IL, USA) was used to analyze the data. The mean ± SD was used to express the results. Analysis of variance (ANOVA) and the least significant difference (LSD) test were used to assess group differences. *P*-values below 0.05 were regarded as statistically significant.

## Results

### METRNL impact on total and differential leukocyte counts in BAL fluid

**Fig. 1:**
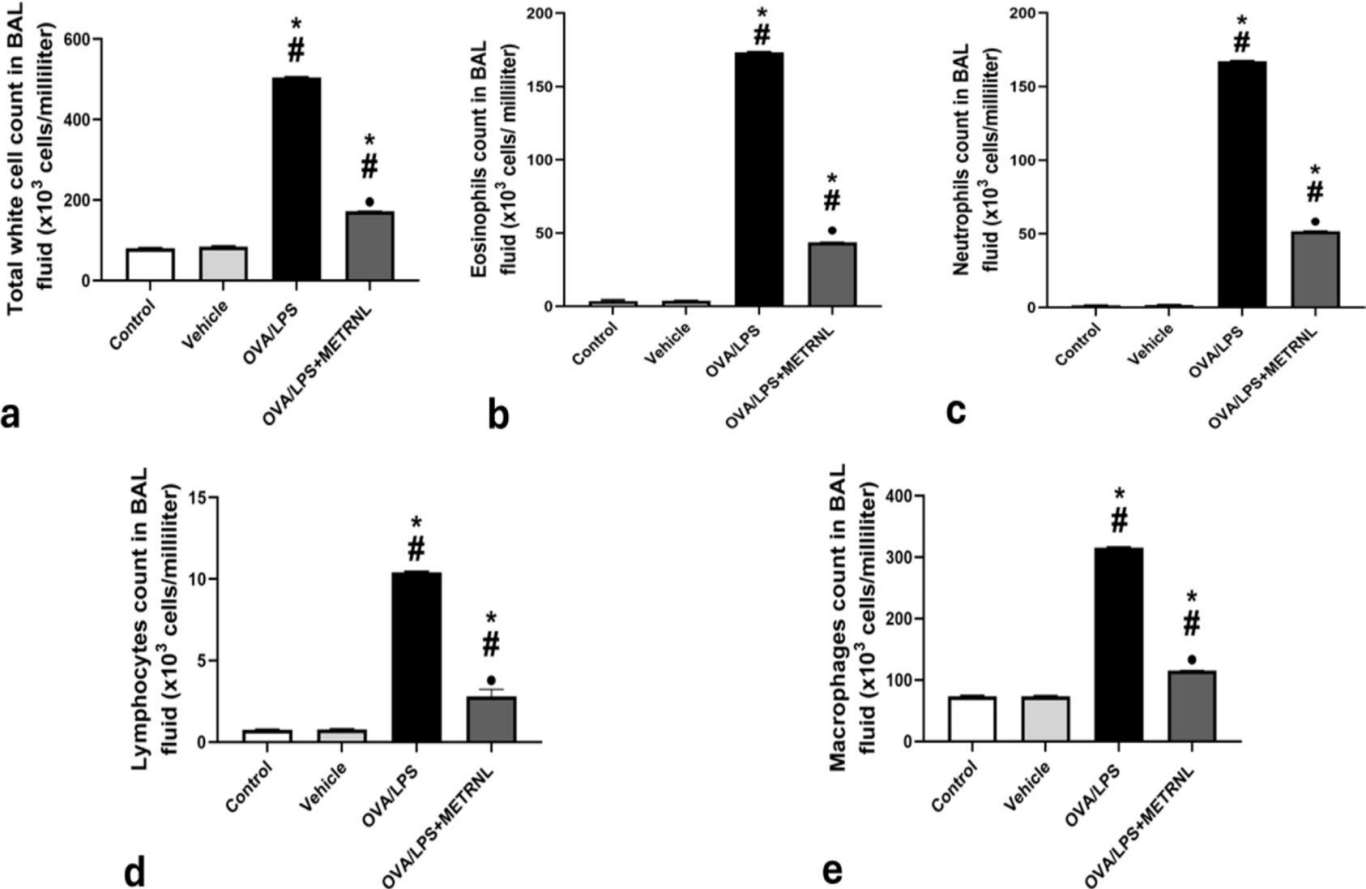
BAL fluid total and differential leucocytic count in different studied groups: **a** total white cell count in BAL fluid (× 10^4^ cells/ml); **b** eosinophil count in BAL fluid (× 10^4^ cells/ml); **c** neutrophil count in BAL fluid (× 10^4^ cells/ml); **d** macrophage count in BAL fluid (× 10^4^ cells/ml); **e** lymphocyte count in BAL fluid (× 10^4^ cells/ml). * *P* < 0.05, significant compared to the control group. # *P*< 0.05, significant compared to the vehicle group. ● *P *< 0.05, significant compared to the OVA/LPS group

The total and differential leukocyte counts in BAL fluid showed no significant changes between the control group (80.4 × 10^3^ ± 0.54 cells/mL, 3.5 × 10^3^ ± 0.85 eosinophils, 1.5 × 10^3^ ± 0.052 neutrophils, 73.6 × 10^3^ ± 0.54 macrophages, 0.076 × 10^3^ ± 0.008 lymphocytes) and the vehicle group (84.3 × 10^3^ ± 0.87 cells/mL, 3.8 × 10^3^ ± 0.12 eosinophils, 1.8 × 10^3^ ± 0.06 neutrophils, 73.4 × 10^3^ ± 0.42 macrophages, 0.78 × 10^3^ ± 0.008 lymphocytes). However, significant increases (*P* < 0.05) in total and differential leukocyte counts were observed in the OVA/LPS group (504.3 × 10^3^ ± 1.42 cells/mL, 173.3 × 10^3^ ± 0.34 eosinophils, 167.3 × 10^3^ ± 0.26 neutrophils, 315.2 × 10^3^ ± 0.90 macrophages, 10.4 × 10^3^ ± 0.05 lymphocytes) and the OVA/LPS + METRNL group (171.8 × 10^3^ ± 0.82 cells/mL, 43.6 × 10^3^ ± 0.28 eosinophils, 51.8 × 10^3^ ± 0.35 neutrophils, 115.2 × 10^3^ ± 0.44 macrophages, 2.8 × 10^3^ ± 0.43 lymphocytes) in contrast to the vehicle and control groups. However, when compared to the OVA/LPS group, the OVA/LPS + METRNL group’s total and differential leukocyte counts were significantly lower (*P* < 0.05) (Fig. 1).

### METRNL impact on oxidative stress markers

There was no discernible difference in the pulmonary SOD levels between the vehicle and control groups (26.95 ± 1.31 vs. 27.50 ± 1.18 U/g.tissue, respectively). In contrast to the control and vehicle groups, the OVA/LPS group showed a significant (*P* < 0.05) drop in SOD levels (13.73 ± 0.77 U/g.tissue).

SOD levels in the OVA/LPS + METRNL group were 21.68 ± 0.52 U/g.tissue, considerably lower (*P* < 0.05) than in the vehicle and control groups. However, the OVA/LPS + METRNL group showed significantly higher SOD levels (*P* < 0.05) than the OVA/LPS group.

**Fig. 2:**
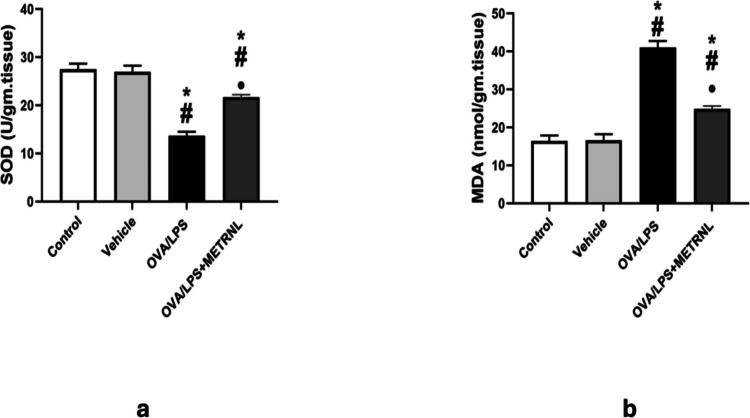
Oxidative stress markers in different studied groups: **a** SOD (U/g.tissue); **b** MDA (nmol/g.tissue). * P< 0.05, significant compared to the control group. # *P*< 0.05, significant compared to the vehicle group. ● *P*< 0.05, significant compared to the OVA/LPS group

A insignificant difference was observed in the mean pulmonary MDA levels between the control and vehicle groups (16.48 ± 1.40 vs. 16.65 ± 1.57 nmol/g.tissue, respectively). However, the OVA/LPS group showed a significant increase (*P* < 0.05) in MDA levels (41.07 ± 1.69 nmol/g.tissue) compared to both control and vehicle groups. In the OVA/LPS + METRNL group, MDA levels were significantly elevated (*P* < 0.05) to 24.87 ± 0.77 nmol/g.tissue when compared to the control and vehicle groups. Nevertheless, the OVA/LPS + METRNL group showed a significant reduction (*P* < 0.05) in MDA levels compared to the OVA/LPS group (Fig. 2).

### METRNL impact on inflammatory mediators

**Fig. 3:**
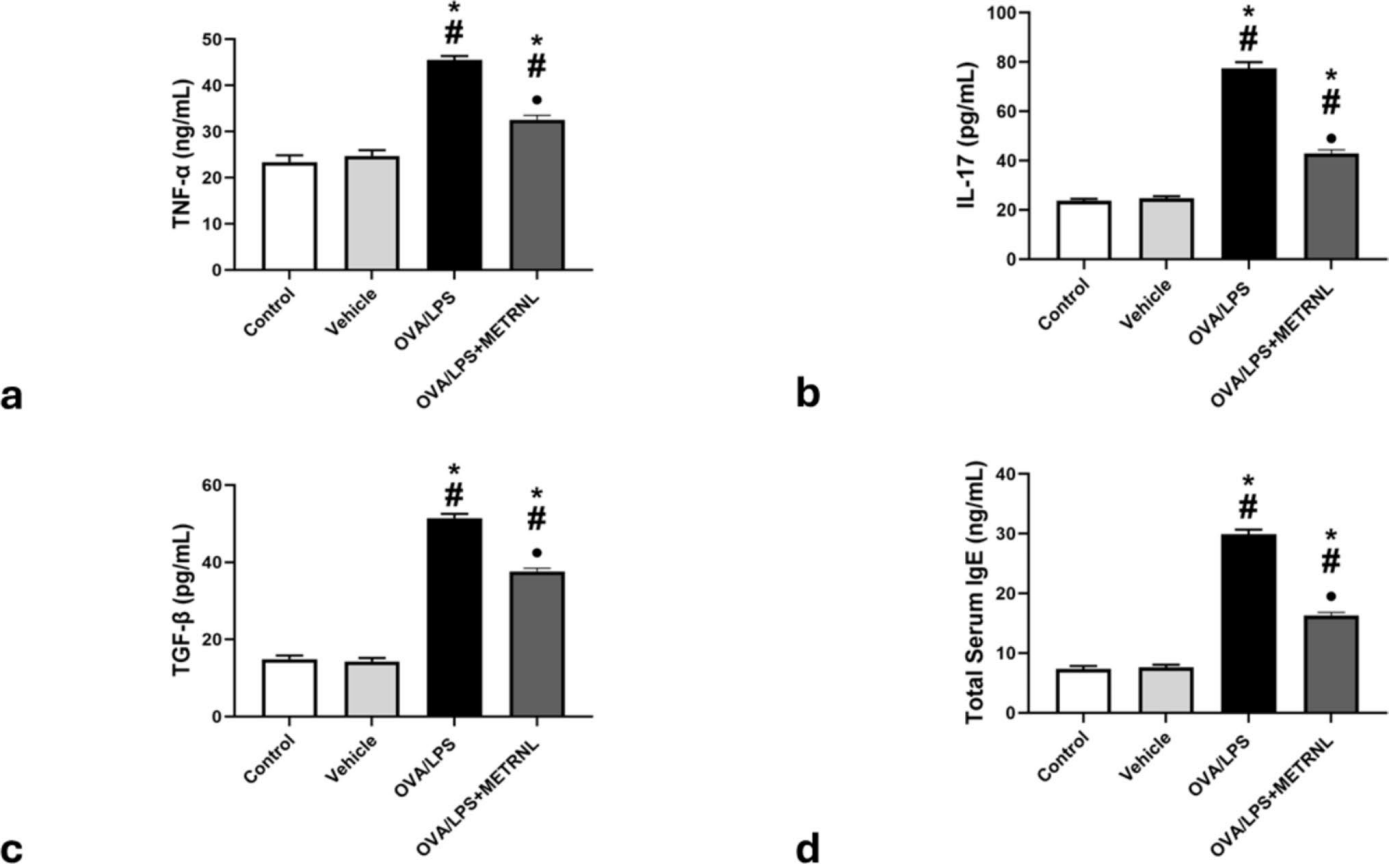
The inflammatory markers and serum IgE in different studied groups: **a** TNF-α (ng/mL); **b** IL-17 (pg/mL); **c** TGF-β (pg/mL); **d** total serum IgE (ng/mL). * *P* < 0.05, significant compared to the control group. # *P *< 0.05, significant compared to the vehicle group. ● *P *< 0.05, significant compared to the OVA/LPS group

The vehicle group (24.73 ± 1.24 ng/mL, 24.64 ± 0.94 pg/mL, 14.28 ± 0.90 pg/mL) and the control group (23.40 ± 1.44 ng/mL, 23.60 ± 0.86 pg/mL, 14.88 ± 0.92 pg/mL, respectively) did not significantly differ in their pulmonary levels of inflammatory mediators (TNF-α, IL-17, and TGF-β). In contrast to the control and vehicle groups, the OVA/LPS group showed a substantial increase (*P* < 0.05) in these mediators (45.50 ± 0.91 ng/mL, 77.48 ± 2.44 pg/mL, 51.42 ± 1.11 pg/mL, respectively). These inflammatory mediators were substantially higher (*P* < 0.05) in the OVA/LPS + METRNL group than in the control and vehicle groups (32.58 ± 0.92 ng/mL, 42.90 ± 1.49 pg/mL, 37.62 ± 0.84 pg/mL, respectively), but significantly lower in the OVA/LPS group (Fig. 3a, b, c).

### METRNL impact on serum IgE

Likewise, there was no discernible difference in serum IgE levels between the vehicle and control groups (7.58 ± 0.47 ng/mL and 7.33 ± 0.50 ng/mL, respectively). In contrast to the control and vehicle groups, IgE levels were considerably higher (*P* < 0.05) in the OVA/LPS group (29.92 ± 0.76 ng/mL) and the OVA/LPS + METRNL group (16.30 ± 0.51 ng/mL). Crucially, serum IgE levels in the OVA/LPS + METRNL group were significantly lower (*P* < 0.05) than in the OVA/LPS group (Fig. 3d).

### METRNL impact on IKK, IκB, and NF-κβ gene expression

The mRNA expression levels of IKK, IκB, and NF-κβ proteins in the vehicle group (0.99 ± 0.004, 0.97 ± 0.043, and 0.99 ± 0.001 did not significantly differ (*P* > 0.05) from the control group. In contrast, the OVA/LPS group exhibited significant upregulation (*P* < 0.05) in the mRNA expression of IKK and NF-κB (6.26 ± 0.40 and 4.45 ± 0.12, respectively) and the OVA/LPS + METRNL group (3.98 ± 0.12; 2.60 ± 0.12, respectively) were significantly upregulated (*P* < 0.05), compared to consistent values in the control group. OVA/LPS + METRNL group mRNA gene expression of the IKK and NF-κβ proteins was significantly downregulated (*P* < 0.05) compared to consistent values in the OVA/LPS group.

**Fig. 4:**
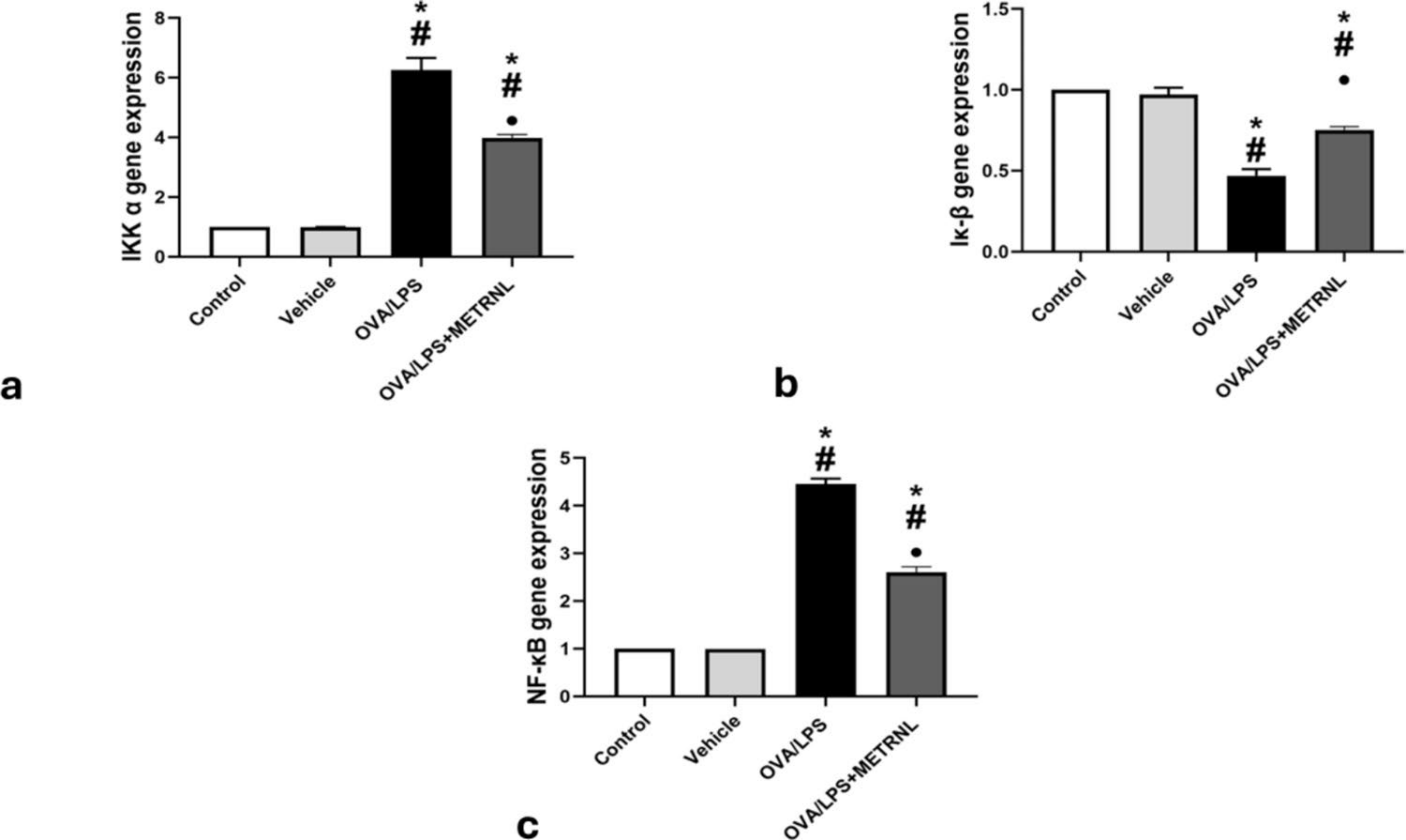
Pulmonary IKK/IκB/NF-κB signaling pathway rt PCR gene expression in different studied groups **a** IKK α, **b** Iκ-β, and **c** nuclear factor-kappa beta NF-κB. * *P *< 0.05, significant compared to the control group. # *P *< 0.05, significant compared to the vehicle group. ● *P* < 0.05, significant compared to the OVA/LPS group

Furthermore, the mRNA expression of IκB was significantly downregulated (*P* < 0.05) in both the OVA/LPS group (0.47 ± 0.040) and the OVA/LPS + METRNL group (0.75 ± 0.023) compared to the control group (Fig. 4).

### Histological results

#### Hematoxylin and eosin staining

**Fig. 5:**
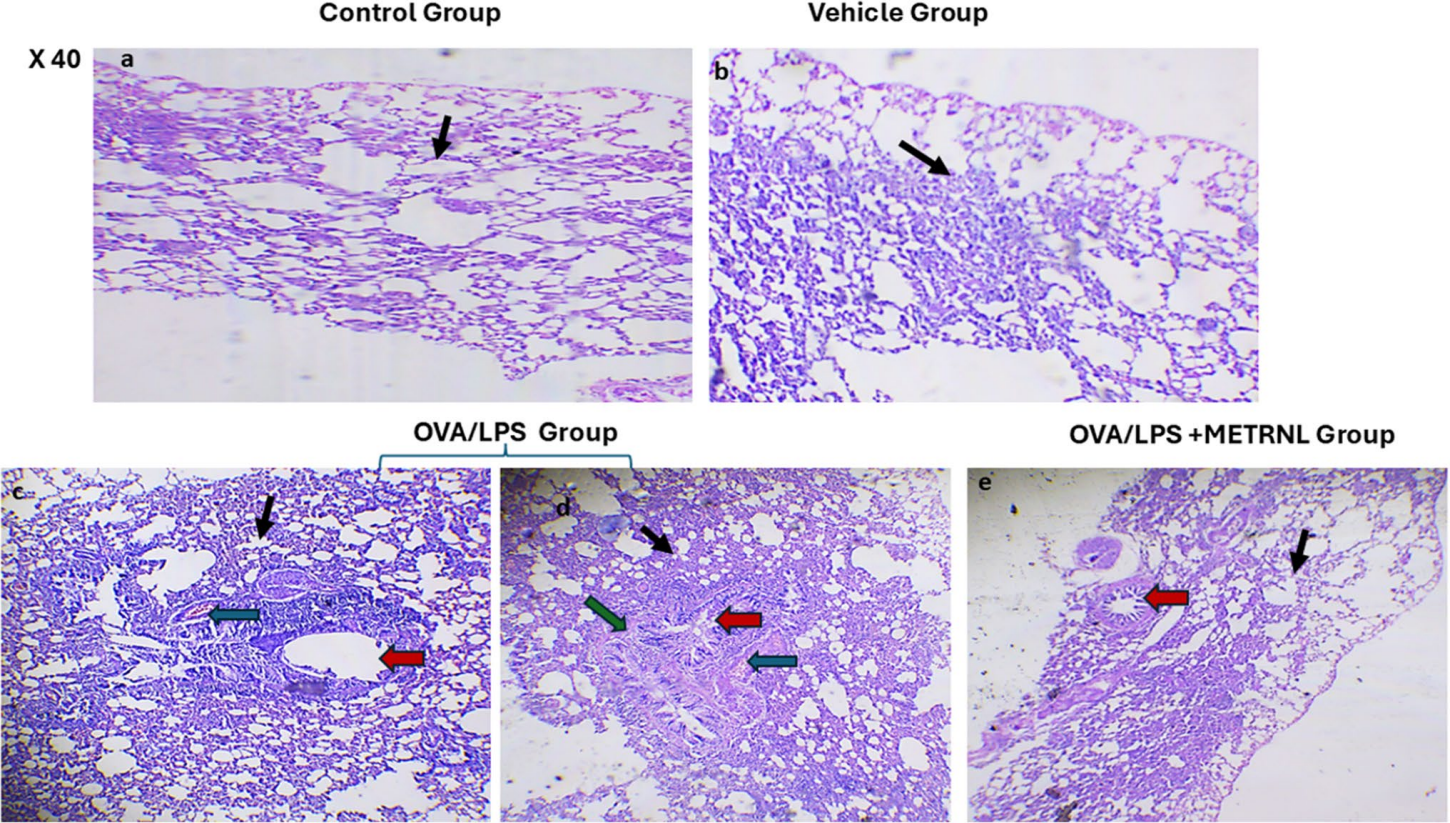
H&E-stained lung sections in the studied groups (H&E 40 ×): **a**,**b** photomicrographs of the vehicle and normal control groups demonstrating normal lung architecture. **c**,**d** Photomicrographs of the ovalbumin plus lipopolysaccharide group demonstrated noticeable distortion in the lung architecture, with bronchiolar and alveolar narrowing and wall thickening demonstrating prominent inflammatory infiltrates and vascular congestion. **e** A photomicrograph of ovalbumin plus lipopolysaccharide + METRNL group showing marked improvements in the lung tissue. Note: Bronchial wall, red arrows; alveoli, black arrows; blood vessels, blue arrow; inflammatory infiltrates, green arrows.

Sections from the control and vehicle groups showed normal lung architecture (Fig. 5a and b). In contrast, the OVA/LPS group exhibited significant structural alterations, including inflammatory cell infiltrates, vascular congestion, alveolar and bronchiolar narrowing, and thickened walls (Fig. 5c and d). These modifications were significantly improved by METRNL (Fig. 5e).

#### Morphometric and statistical results

### Caspase-3 intensity percentage

**Fig. 6:**
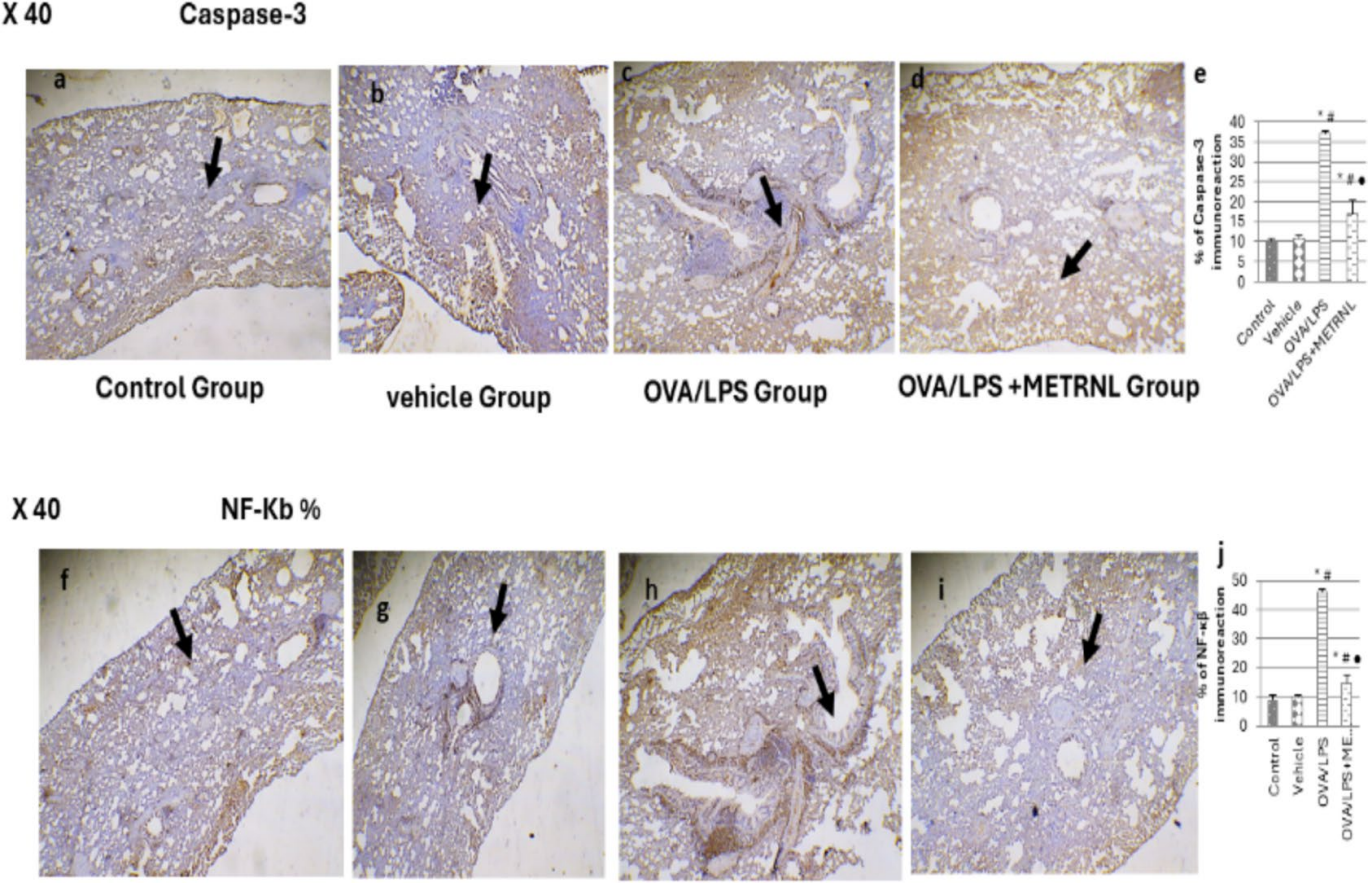
** a**, **b**, **c**,**d** Photomicrographs representing caspase-3-stained lung sections. **a**,**b** The control and vehicle groups showing negative immunostaining for caspase 3. **c** The OVA/LPS group showed an intense brown color distributed throughout the damaged area in the lung tissue. **d** The OVA/LPS + METRNL group showed faint cytoplasmic immunostaining for caspase-3 in lung tissue (caspase-3 40 ×). **f**,**g**,**h**,**i** Photomicrographs that represent NF-κβ-stained lung sections. **f**,**g** The control and vehicle groups showed negative cytoplasmic immunostaining for NF-κβ. h The OVA/LPS group showed a positive reaction to NF-κβ with an intense brown color distributed throughout the damaged areas. **i ** The OVA/LPS + METRNL group showing faint cytoplasmic immunostaining for NF-κβ (NF-κβ 40 ×). **e**,**j** Impact of METRNL on caspase-3 and NF-κβ stains. Data expressed as mean ± SD ( n = 10). ANOVA has been utilized to make group comparisons.

The mean caspase-3 intensity did not differ significantly between the vehicle and control groups (10 ± 0.74 vs. 11 ± 0.53, respectively, *P* > 0.05). The OVA/LPS group’s caspase-3 intensity was significantly higher than that of the vehicle and control groups (37 ± 0.54, *P* < 0.05). Caspase-3 intensity was substantially lower in the OVA/LPS + METRNL group than in the OVA/LPS group (17 ± 0.54, *P* < 0.05). However, the intensity was still much higher than in the vehicle and control groups (*P* < 0.05) (Fig. 6e).

### NF-κβ intensity percentage

Regarding NF-κβ intensity, there was not a significant distinction between the vehicle and control groups (9 ± 1.39 vs. 10 ± 0.45, respectively, *P* > 0.05). The NF-κβ intensity was substantially higher in the OVA/LPS group than in the vehicle and control groups (46 ± 0.94, *P* < 0.05). NF-κβ intensity was considerably higher in the OVA/LPS + METRNL group than in the control and vehicle groups (*P* < 0.05). In contrast, it was significantly lower in the OVA/LPS group (15 ± 2.7, *P* < 0.05) (Fig. 6j).

### Discussion

Millions of people worldwide have asthma, a complicated inflammatory illness. In COPD and other disorders, METRNL, a recently identified secretory cytokine /adipokine/ myokine, has demonstrated protective properties. (Baht et al. 2020; Hu et al. 2020; Kerget et al. 2020). However, its specific role in allergic asthma remains unclear.

Research by Lee et al. demonstrated that immunization with OVA is commonly used to induce eosinophilic airway allergy in experimental models, triggering oxidative stress and an inflammatory response mediated by T-helper 2 cells. This method simulates asthma characteristics such as airway inflammation, eosinophil infiltration, airway hyperresponsiveness, and increased mucus secretion (Lee et al. 2010).

The combination of OVA and LPS (a toll-like receptor agonist), as shown by Kim et al. (2007), produces Th1-mediated neutrophilia and increases cytokine levels, making it useful in experimental allergic airway disease models.

The current study demonstrated that OVA/LPS induced significant oxidative stress, as indicated by a substantial rise in MDA levels and a reduction in SOD activity in lung tissue, which aligns with previous research (Nader 2015).

These oxidative stress markers (MDA and SOD) can indicate the severity of allergic inflammation (Ammar et al. 2022). SOD, a crucial antioxidant enzyme, converts ROS (reactive oxygen species) into less harmful substances, playing a key role in defending against oxidative stress (Aldini et al. 2011; Zinellu et al. 2016). Reduced SOD activity has been observed in asthma patients (Brown et al. 2007). A study regarded OVA/LPS as crucial reference protein allergens widely employed in asthma models due to their role in modulating T-cell immune responses (Thakur et al. 2019).

METRNL is a novel secreted protein homologous. Li et al. (2023) claimed that METRNL exerts a pleiotropic ameliorative effect on oxidative stress, inflammation, immune response, and metabolism. In this study, METRNL treatment significantly decreased MDA levels while elevating SOD activity, demonstrating its protective effects.

Inflammatory mediators (TNF-α, IL-17, and TGF-β) were significantly elevated in the OVA/LPS group, consistent with the results of Abbasnia et al. (2024). ROS, produced during inflammation, can amplify inflammatory reactions, establishing a link between oxidative stress and inflammation in allergic airway disease. The increase in TNF-α, a mediator of allergic lung inflammation, and IL-17, linked to severe asthma through the upregulation of Th2-related cytokines and the downregulation of Th1 cells, further illustrates the inflammation present in asthma (Liu et al. 2013).

Different cell types generate TNF-α because of allergic pulmonary inflammation, such as mast cells (Lee et al. 2010). Moreover, severe asthma has consistently been associated with increased levels of IL-17 in the airway. However, the causality of this relationship is uncertain and requires additional investigation (Hynes and Hinks 2020).

METRNL significantly decreased inflammatory markers TNF-α, IL-17, and TGF-β in asthma compared to the OVA/LPS group, which agreed with previous findings (Brightling et al. 2008; Jung et al. 2018).

This study’s novelty was in the antioxidant and anti-inflammatory effect of METRNL on the allergic airway rat model.

The study highlights the novel antioxidant and anti-inflammatory effects of METRNL in an allergic airway rat model. METRNL significantly decreased inflammatory markers and oxidative stress while improving lung histopathology. Similar findings were observed by Liu et al. (2024), where METRNL was shown to reduce inflammation in sepsis by activating the NF-κB, IKK, and phosphorylation of IκBα (Hu et al. 2021). METRNL was also found to lower pro-inflammatory cytokines, suggesting its role in macrophage recruitment and immune regulation (Chen et al. 2023).

METRNL was crucial in reducing airway hyperreactivity by suppressing the activity of dendritic cells responsible for regulating adaptive immune responses through enhancing AMPK or PPAR-ɤ signaling pathways, which reduces the inflammatory response via NF-κB signaling (Gao et al. 2022).

The histopathological examination confirmed these results, revealing clear evidence of OVA/LPS-induced bronchial asthma. The macroscopic picture demonstrated significant pathological alterations in the lung structure, including narrowing of the bronchioles and alveoli, thickening of the walls, and the presence of pronounced inflammatory infiltrates with vascular congestion and thickened muscular layer. The findings of this study agreed with the results reported by Elaidy et al. (2018) and Azman et al. (2021). These investigations also observed significant infiltration of inflammatory cells and congestion in the blood vessels of lung tissues generated by OVA/LPS.

METRNL treatment improved lung tissue damage, aligning with research by Huang et al. (2024), who demonstrated METRNL’s ability to reduce allergic inflammation by suppressing immune cells and inflammation-associated genes.

In addition, OVA/LPS increased serum IgE levels, which were significantly reduced with METRNL treatment. IgE production is triggered by Th2 cells and cytokines like IL-4 and IL-13 through histamine release from sensitized mast cells. These cytokines are responsible for stimulating B cells to produce IgE antibodies with activation and recruiting eosinophils (Leigh et al. 2004; Possa et al. 2013).

METRNL’s antioxidant properties likely suppressed IgE production by preventing ROS-induced inflammatory responses, corroborating recent research by Chen et al. METRNL effectively reduced the occurrence of ferroptosis generated by LPS by modulating the SIRT1-P53-SLC7A11 signaling pathway. Additionally, METRNL was found to lower the severity of OVA/LPS-induced lung injury (Chen et al. 2023).

The present study also demonstrated increased eosinophil recruitment in the BALF and lung tissue of asthmatic rats exposed to OVA/LPS, consistent with prior research (Sun et al. 2010). Eosinophil infiltration is driven by IL-5 and eotaxin, key players in asthma development (Ribeiro-Filho et al. 2013). Eotaxin is a highly effective substance that attracts eosinophils. It is found in large amounts in the respiratory epithelium after exposure to OVA/LPS, which increases eosinophils in the lungs (Lilly et al. 1997).

In research performed by Kumari et al., the histological analysis of the lung revealed a significant presence of leukocytes and heightened growth of epithelial cells. It enhanced smooth muscle thickening caused by the injection of OVA/LPS, which could be attributed to the cumulative impact of allergens on eosinophils and neutrophils. Consequently, rats exposed to OVA/LPS inhibited significant narrowing of the bronchial airways, accompanied by inflammation character, resized by eosinophils, neutrophils, and lymphocytes (Kumari et al. 2015).

In line with the findings of Yao et al. regarding METRNL’s function in controlling immunological responses, METRNL therapy markedly decreased eosinophilic and lymphocytic infiltration. Moreover, macrophages ought to be necessary for maintaining the synthesis of various cytokines and chemokines (Yao et al. 2021). Thus, this represents an “amplification loop” that promotes the activation of macrophages. Ushach et al. (2018) reported that METRNL has a depressant effect on the eosinophil-dependent increased interleukins.

At the molecular level, OVA/LPS upregulated the expression of IκB kinase and NF-κB while downregulating IκB expression, contributing to inflammation. These changes are associated with fibrosis and inflammation in asthma. This was in line with a study by Dong and Yuan (2018).

METRNL treatment downregulated the NF-κB pathway, reducing inflammation, which aligns with the findings of Liu et al. (2024).

As inflammation is one of the core pathologies of asthma, recently, it has been found to be linked with the modulation of the IKK/IκB/NF-κB pathway (Liu et al. 2024). After IKK bound to stimuli, such as cytokines, growth factors, stressors, and mitogens, the IKK multi-subunit complex became phosphorylated. As a result, the IκB became phosphorylated (Sun 2017). When an inhibitor of NF-κβ is phosphorylated, it is separated and then translocated to the nucleus, and this induces the production of pro-inflammatory mediators (Reynolds et al. 2010). The NF-κB pathway plays a critical role in regulating pro-inflammatory gene expression and T-cell activity, making it a key target for anti-inflammatory therapies (Saad 2021).

This study is among the first to investigate METRNL’s role in modulating the IKK/IκB/NF-κB pathway in asthma. The results suggest that METRNL could be a promising therapeutic option for reducing inflammation in asthma by targeting this pathway.

## Conclusion

The combination of OVA and LPS induced severe asthma phenotypes characterized by significant inflammation, increased ROS production, and elevated IgE levels in BALF. Additionally, OVA/LPS exposure altered the pro-inflammatory pathway involving IKK, IκB, and NF-κB proteins—markers that were studied in bronchial asthma for the first time in this context. Histological analysis confirmed apoptosis through significant upregulation of the caspase-3 protein.

The newly administered METRNL displayed promising antioxidant, anti-inflammatory, and antiapoptotic effects in this OVA/LPS-induced bronchial asthma model. METRNL significantly reduced eosinophilia, IgE levels, and WBC infiltration in BALF while also modulating the disrupted pro-inflammatory IKK, IκB, and NF-κB pathways. These findings reveal the previously unappreciated protective role of METRNL in mitigating inflammation in allergic asthma, suggesting it could serve as a novel therapeutic strategy by targeting the NF-κB, IκB, and IKK pathways for asthma treatment.

## Data Availability

Upon reasonable request, the corresponding author will provide the datasets used and/or analyzed in the current study.

## Declarations

### Use of artificial intelligence

The authors declare that they have not used artificial intelligence (AI)-tools for writing the manuscript.
